# Differential Inductive Sensing System for Truly Contactless Measuring of Liquids′ Electromagnetic Properties in Tubing

**DOI:** 10.3390/s21165535

**Published:** 2021-08-17

**Authors:** Marc Berger, Anne Zygmanowski, Stefan Zimmermann

**Affiliations:** Department of Sensors and Measurement Technology, Institute of Electrical Engineering and Measurement Technology, Leibniz University Hannover, 30167 Hannover, Germany; zygmanowski@geml.uni-hannover.de (A.Z.); zimmermann@geml.uni-hannover.de (S.Z.)

**Keywords:** differential transformer, inductive conductivity measurement, contactless measurement, tubing guides sample, hose guides sample, dialysis treatment, sodium monitoring, differential inductive sensor, PCB coil

## Abstract

Certain applications require a contactless measurement to eliminate the risk of sensor-induced sample contamination. Examples can be found in chemical process control, biotechnology or medical technology. For instance, in critically ill patients requiring renal replacement therapy, continuous in-line monitoring of blood conductivity as a measure for sodium should be considered. A differential inductive sensing system based on a differential transformer using a specific flow chamber has already proven suitable for this application. However, since the blood in renal replacement therapy is carried in plastic tubing, a direct measurement through the tubing offers a contactless method. Therefore, in this work we present a differential transformer for measuring directly through electrically non-conductive tubing by winding the tube around the ferrite core of the transformer. Here, the dependence of the winding type and the number of turns of the tubing on the sensitivity has been analyzed by using a mathematical model, simulations and experimental validation. A maximum sensitivity of 364.9 mV/mol/L is measured for radial winding around the core. A longitudinal winding turns out to be less effective with 92.8 mV/mol/L. However, the findings prove the ability to use the differential transformer as a truly contactless sensing system.

## 1. Introduction

To avoid possible contamination of a sample induced by a sensing system, in some applications it is essential to perform contactless measurements. An example of such an application is the continuous in-line monitoring of blood conductivity as a measure of the sodium concentration of blood serum [1,2,3,4,5,6]. Such monitoring is particularly useful for critically ill patients in intensive care units with severe dysnatremia [7,8,9]. These patients often receive continuous renal replacement therapy. One of the objectives of that therapy is the normalization of sodium towards the physiological level [10]. However, a preceding severe dysnatremia requires a much slower rate of normalization compared to intermittent dialysis patients [11,12]. Hence, a patient-individualized therapy should be used requiring continuous monitoring of the patient′s sodium concentration [13,14,15]. Otherwise, serious side effects such as central pontine myelinolysis may occur [16]. To avoid further stress on the patient by drawing blood samples and to enable a continuous monitoring, this parameter should be measured in-line. Since the blood inside the tubing of the extracorporeal circuit is returned to the patient after passing the dialyzer [17], a contactless measurement offers the inherent advantage of preventing contamination caused by the sensor. However, high-frequency measurement systems operating in the microwave range are not suitable for measuring the sodium serum concentration. In order to measure the sodium serum concentration in blood, the measurement frequency must remain clearly below the beta-dispersion. Beta-dispersion is at about 1 MHz. In measuring systems that operate above this frequency, the whole blood conductivity is additionally affected by the high concentration of intracellular potassium in red blood cells. Thus, solely measuring the sodium concentration (neglecting the low concentrated electrolytes in extracellular liquid) in the microwave region is not possible [3,18,19]. Further examples for requiring a contactless sensing system can also be found in biological and chemical process monitoring [20,21]. For instance, the dielectric properties (permittivity and dielectric losses) of a solution can be used to monitor cell growth in liquids [22,23]. As contamination of the sample would endanger the cultivation process, the sensor usually requires extensive sterilization. With a contactless measuring system, this time-consuming and cost-intensive step is eliminated for the sensor system [24,25,26].

One example of a contactless measuring system is the capacitively coupled contactless conductivity detection (C^4^D), enabling capacitive measuring through a special fused-silica capillary with a small cross-section in the micrometer range [27,28,29]. Here, two electrodes are capacitively coupled with sample and capillary as dielectrics. A change in the conductivity of the sample within the capillary changes the impedance between the electrodes. Consequently, the electrode current can be used to determine the sample conductivity. However, due to the small cross-section of the special fused-silica capillary, it is not possible to measure in-line in certain applications where a higher flow rate is required as e.g., in dialysis treatment with about 100–500 mL/min [30,31]. A further drawback of this method is a frequency-dependent electrode polarization [32] and a strongly dominating impact of the capillary wall on the total impedance as mentioned in [33]. This was also closely investigated in [25]. It was found that capacitive measuring principles are less suitable for contactless determination of the sample properties when the sample is contained in materials with low dielectric constant *ε*′, e.g., the tubing of the extracorporeal circuit in dialysis treatment are usually made of polyvinyl chloride (PVC) having a relative dielectric constant *ε_r_*′ of about 3–5 [34,35]. Therefore, inductive sensing systems were identified in [25] as better suited for such applications.

Inductive conductivity sensors are available in different configurations, for example with one or two coils [26,36]. The disadvantage of these sensors is an output signal with a strong offset [24]. The primary magnetic field, generated by the excited primary coil, induces a voltage into the detecting coil and overlays the measuring signal. Therefore, we use a differential inductive approach based on a differential transformer. A differential transformer typically consists of three coils located on a ferrite core. The middle coil is the primary coil and the outer coils are the secondary coils. The secondary coils are connected in series and have the same inductance but different winding directions. If the ferrite core can be moved relative to the coils, a linear variable differential transformer is formed. Typical applications of a linear variable differential transformer are precise displacement, force, speed or pressure measurements [37,38,39,40,41].

**Figure 1:**
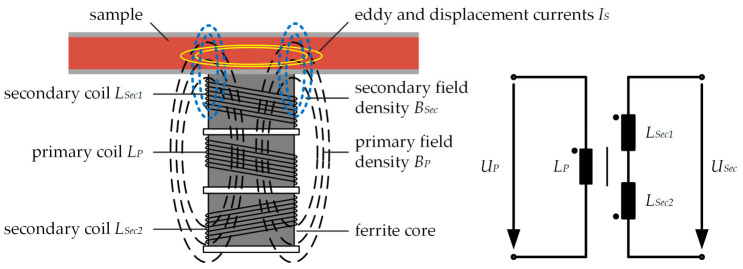
Schematic illustration of a differential transformer for measuring the electromagnetic properties of a sample. The differential transformer consists of three coils located on a ferrite core. The connection of the coils is illustrated in the equivalent circuit of the unloaded differential transformer on the right-hand side.

However, in our case, the ferrite core is fixed and cannot be moved relative to the coils. Figure 1 schematically depicts the differential transformer for measuring the electromagnetic properties (conductivity, permittivity and dielectric losses) of a sample.

By exciting the primary coil *L_P_* with an AC voltage *U_P_*, the primary magnetic field density *B_P_* is generated. *B_P_* penetrates both, the secondary coils *L_Sec1_* and *L_Sec2_* as well as the sample. Since both secondary coils *L_Sec1_* and *L_Sec2_* have the same inductance and are arranged symmetrically to *L_P_*, a voltage of the same magnitude is induced into each coil. However, due to different winding directions of *L_Sec1_* and *L_Sec2_*, as also indicated by the dot in the equivalent circuit in Figure 1, the sing of the induced voltage is opposite. Thus, the strong primary magnetic field causes no output voltage *U_Sec_*. Hence, the offset of *U_Sec_* is low or even zero for identical secondary coils, resulting in the major advantage over non-differential approaches. This enables an easier detection of the weaker secondary magnetic field density *B_Sec_*, caused by the induced eddy and displacement currents *I_S_* inside the sample. *I_S_* in turn is induced by *B_P_* and depends on the sample conductivity *κ*, its dielectric constant *ε′* and dielectric losses *ε*″. As the sample is placed closer to the upper secondary coil *L_Sec1_* compared to *L_Sec2_*, a higher voltage is induced into *L_Sec1_* due to the secondary field density *B_Sec_*, resulting in an output voltage *U_Sec_*. Since the induced eddy and displacement current density is a circular rotating field and no electrodes are needed for the measurement, there are no electrode polarization effects at low measurement frequencies as for example with C^4^D. According to [25] the output voltage *U_Sec_* can be separated into an real and imaginary part as

(1) $$U_{Sec}=U_{P}\left(\omega^{2}K\varepsilon^{\prime }-j\omega K\left(\kappa +\omega \varepsilon^{\prime \prime }\right)\right),$$

whereby the imaginary part is indicated by the imaginary unit *j* and depends on the conductivity *κ* and dielectric losses *ε″*. The real part depends on the dielectric constant *ε*′, where *ω* is the angular frequency of *U_P_* and *K* describes the magnetic coupling including the inductance of *L_P_* and the mutual inductance between both, the sample and *L_P_* as well as the sample and the secondary coils. As the dielectric losses can be neglected if the measuring frequency does not match a resonance in polarization, Equation (1) enables distinction between electrical and dielectric properties [19,42].

We already used such differential transformer successfully in [4,9] for continuous in-line monitoring of the blood conductivity as a measure for the sodium concentration using reconfigured human packaged red blood cells as sample in a preclinical investigation. Although the differential transformer uses a contactless measuring principle, a specially designed flow chamber was necessary for this investigation [4,9]. For in-line monitoring of the blood, the flow chamber must meet high requirements of hemo and biocompatibility. For example, the flow rate of the blood, carried in the tubing of the extracorporeal circuit during dialysis treatment, must remain nearly constant when entering the flow chamber. Therefore, the flow cross-section of the flow chamber is subject to certain restrictions and may not be varied arbitrarily. However, not only the cross-section itself is important, but also the geometry in general. Turbulent flow and vortexes can occur at the transition points between flow chamber and tubing [43,44]. In order to avoid the risk of hemolysis of the blood due to shear stress, turbulent flows and vortexes has to be avoided. In addition, the chamber must meet the requirements of hemo and biocompatible materials, as it is in direct contact with the blood. All the aforementioned specifications make the design process highly complex and sometimes contradicts the requirements for a highly sensitive sensing system since, e.g., the radius and the height of the flow chamber cannot be changed arbitrarily [45]. Furthermore, disposables are often used in medical technology to prevent elaborate sterilization. This associates the flow chamber with higher costs.

Since we have already investigated the general effects of the sample geometry on the sensitivity of a differential transformer in [45], this paper is a direct continuation of the previous work. The objective is to develop and investigate a new approach of a truly contactless method to measure the electromagnetic properties of samples in electrically non-conductive tubing without the need for a special designed flow chamber. Therefore, the sample is analyzed directly through the tubing. Hence, turbulent flows and vortexes at the transition points between the tubing and the flow chamber can be avoided in applications where the sample is carried in tubing anyway, such as in dialysis treatment, making a specific flow chamber obsolete. Thus, at first it has to be verified whether it is possible to measure in-line through an electrically non-conductive tubing. Afterwards, the effect of different tubing settings on sensitivity and precision will be investigated.

## 2. Materials and Methods

This section describes the materials and method used in this work. Section 2.1 focuses a real differential transformer used in all experiments. In addition, the setup of the experiments as well as the sample solutions used and their concentration range are described here. Section 2.2 addresses the CST-EM Studio simulation model that is used additionally to the experiments.

### 2.1. Printed Circuit Board (PCB)-Differential Transformer and the Experimental Setup

**Figure 2:**
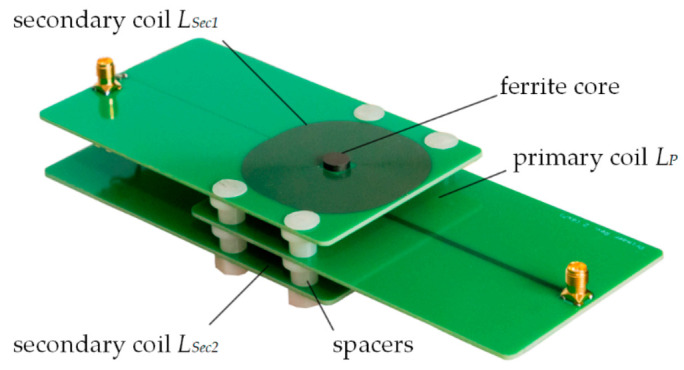
Photography of the used differential transformer made of three printed circuit board (PCB) coils. The differential connection of *L_Sec1_* and *L_Sec2_* is realized via two wires (not seen).

We use a differential transformer consisting of three printed circuit board (PCB) coils located on a ferrite core for the experimental characterization of a sample. This real differential transformer was designed in previous works [9,45]. The PCBs contain 35 µm thick copper tracks forming the coils. Setting the distance *d_PCB_* between each planar PCB to a certain value affects the magnetic coupling between the coil an the sample, which can have positive effects on the sensitivity [9]. Here, *d_PCB_* is set to 8 mm using spacers. Figure 2 shows a photograph of the used PCB differential transformer.

**Figure 3:**
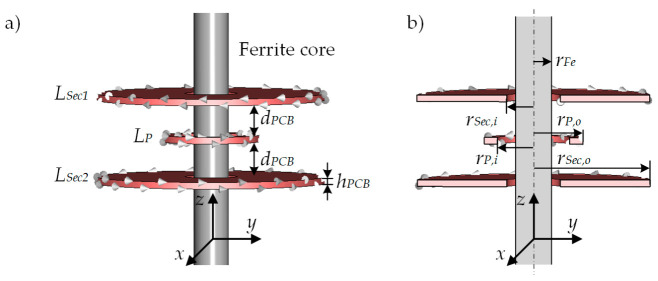
(**a**) Illustration of the CST-EM Studio simulation model of the differential transformer without sample. Small white cones indicate the winding direction of the coils. As can be seen, *L_Sec1_* and *L_Sec2_* have an opposite winding direction. The distance *d_PCB_* between the coils and the height *h_PCB_* of the PCB coils corresponds to the real PCB differential transformer from Section 2.1. (**b**) Cross-section through the *y-z*-plane of the simulation model. The radii of the coils and the radii of the ferrite core *r_Fe_* corresponds to the radii from the experimental PCB differential transformer. The coordinate system is defined with the *z*-axis pointing longitudinally to the ferrite core. The *x*- and *y*-axes run radially to the ferrite core. The relative permeability *µ_r_* of the ferrite core is 300. The number of turns for *L_P_*, *L_Sec1_* and *L_Sec2_* corresponds to the number of turns from Section 2.1. The same applies to the relative permeability *µ_r_* of the ferrite core.

The coils are located on a ferrite core having a radius of 4 mm and a relative permeability *µ_r_* of 300 at the used measuring frequency. The ferrite core is a manganese-zinc K300 core. The imaginary part of the relative permeability (losses) is below 2 in the used frequency range and can therefore be neglected. The length of the core is 200 mm. All PCB coils were realized on a separate 90 mm × 120 mm × 1.5 mm six-layer PCB. The PCBs were made of FR-4. The primary coil *L_P_* is located between the outer coils with a total of 42 turns, giving 7 turns per layer with a track width of 0.3 mm and a clearance of 0.125 mm. The mean coil radius *r_M,P_* is 9.55 mm calculated as the arithmetic mean of the outer coil radius *r_P,o_* = 11 mm and the inner coil radius *r_P,i_* = 8.1 mm. All dimensions are shown in Figure 3. The measured DC resistance of *L_P_* is 3.4 Ω and the measured inductance is 31.9 µH. The secondary coils *L_Sec1_* and *L_Sec2_* are the upper and lower coils, having a total number of 542 turns each on the six-layer board. The track width is 0.1 mm with a clearance of 0.125 mm. The inner coil radius *r_Sec,i_* is 6 mm and an outer coil radius *r_Sec,o_* is 26 mm, resulting in a mean coil radius *r_Sec,M_* of 16 mm. The measured inductances of *L_Sec1_* and *L_Sec2_* are each 23.8 mH with a measured DC resistance of 320 Ω. Via two wires *L_Sec1_* and *L_Sec2_* are connected differentially in series. The primary coil and the upper secondary coil *L_Sec1_* have SMA connectors for the electrical connection. *L_P_* is excited with a voltage of 1 V_PP_ peak to peak at a frequency of *f* = 155 kHz. The frequency was chosen in order to have a sufficient frequency offset from the resonance frequency of about 250 kHz of the secondary coils, as the coils have no inductive characteristics above this frequency.

As a tube, we used a TYGON^®^-ND 100-65 tubing with an inner diameter *D_t,i_* of 4 mm and an outer diameter *D_t,o_* of 5.6 mm carrying the sample along the upper secondary coil *L_Sec1_* [46]. The peristaltic pump Ismatec Ecoline VC-380 pumps the sample from a liquid container into the tubing along the differential transformer and then back into the liquid container [47]. As a sample, we used deionized water (DI-water) with different concentrations of sodium chloride (NaCl). The solution concentration *c* varied in steps: 100 mmol/L, 110 mmol/L, 130 mmol/L, 140 mmol/L and 150 mmol/L. The stepwise increase in concentration was achieved by adding a stock solution (1 mol/L NaCl in DI-Water) in the appropriate amount to the liquid container. To prepare the stock solution, NaCl was purchased for Sigma-Aldrich and dissolved in DI-water. This concentrations range was used in all experiments and covered the pathological concentration range of sodium inside the blood serum, which is of interest for the aforementioned application for continuous renal replacement therapy. Although the concentration generally has a non-linear impact on the sample conductivity [48], it could be shown in [4] that the imaginary part of the output voltage *U_Ses_* of the differential transformer has a linear dependence on the concentration within this narrow range. Considering the absolute value of the sensitivity *S_c_* of the differential transformer with regard to the concentration *c* as input variable, it can be determined from the slope of the linear regression of the concentration-dependent imaginary part of the output voltages *U_Sec_*(*c*) using Equation (2)

(2) $$S_{c}=\frac{d\left|Im\left\{U_{Sec}\left(c\right)\right\}\right|}{dc}$$

### 2.2. Numerical Simulation Model

A CST-EM Studio model was used additionally to the experimental tests. Figure 3a shows the model of the unloaded (without sample) differential transformer. The model represents the differential transformer described above. The primary coil *L_P_* of the simulation model was excited in the same manner as *L_P_* of the real PCB differential transformer in Section 2.1. The secondary coils *L_Sec1_* and *L_Sec2_* were excited with a current of 0 A, corresponding to the condition of an ideal voltage measurement. The voltage induced into the secondary coils can be extracted from CST-EM Studio and divided in real and imaginary parts. CST-EM Studio indicates the winding direction of the coil by small white cones. As can be seen, the winding direction of *L_Sec1_* and *L_Sec2_* is opposite. Thus, the addition of the induced voltages into *L_Sec1_* and *L_Sec2_* gives the output voltage *U_Sec_* of the differential transformer. Figure 3b contains a cross-section in the *y-z*-plane through the simulation model. All radii correspond to those of the PCB differential transformer from the experimental Section 2.1. In contrast to the experimental investigation, the conductivity *κ* of the sample can be easily changed in the simulation model.

As expected from Equation (1), *Im{U_Sec_*} depends linearly on *κ* [9]. The conductivity of the sample varied between 1 S/m and 2 S/m. This allowed the absolute value of the sensitivity *S_κ_* of the differential transformer to be determined in terms of the conductivity *κ* as input variable according to

(3) $$S_{\kappa }=\frac{d\left|Im\left\{U_{Sec}\left(\kappa \right)\right\}\right|}{d\kappa }.$$

For all following sections, the coordinate system is defined as shown in Figure 3. The *z*-axis runs in the longitudinal direction, i.e., along the ferrite core. The *x*- and *y*-axis point in radial direction.

## 3. Results and Discussion

This section investigates a truly contactless measurement method of samples carried in electrically non-conductive tubing. This makes a specific designed flow chamber obsolete and avoids additional issues, such as hemolysis.

The primary magnetic flux density *B_P_* of the primary coil, orientated in the *z*-direction, induces circular eddy and displacement currents *I_S_* into the sample located above *L_Sec1_* [45]. These circular currents rotate around the *z*-axis in the *x-y*-plane or around the ferrite core, respectively. Therefore, it is useful to wrap the tubing at least once around the ferrite core in the *x-y*-plane instead of just passing it in a straight line above *L_Sec1_*. Multiple turns of the tubing around the ferrite core offer two basic winding concepts. The tubing can be wrapped longitudinally to the ferrite core, i.e., a multi-layer winding in *z*-direction with *n_L_* turns, or a planar multi-layer winding with *n_R_* turns in radial direction lateral to the ferrite core is possible, or both. However, the combination is not investigated, as the general findings of the two basic winding options can be transferred. It has been investigated whether multiple turns improve the sensitivity and precision. First, the winding in radial direction with *n_R_* turns will be investigated in Section 3.1 in more detail. After that, Section 3.2 considers the winding in longitudinal direction with *n_L_* turns.

### 3.1. Radial Winding

First, in Section 3.1.1, the behavior of the differential transformer and its dependence on the *n_R_* radial turns is simulated using the CST-EM Studio model presented in Section 2.2. In order to develop a more detailed understanding of this behavior, a mathematical model is subsequently developed in Section 3.1.2, which is validated using the simulations. Finally, Section 3.1.3 presents the experimental investigation of radial winding using the PCB differential transformer from Section 2.1. In addition, the empirical standard deviation is examined in more detail.

#### 3.1.1. Numerical Simulations for Radial Winding

**Figure 4:**
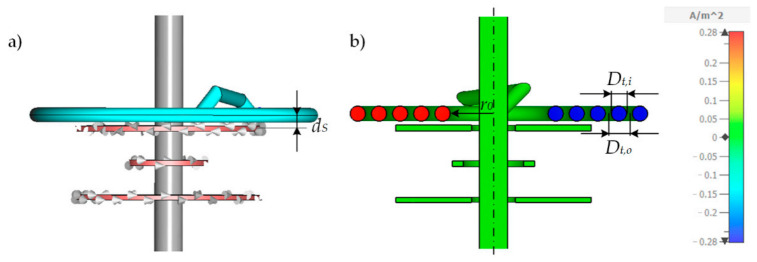
(**a**) Numerical CST-EM Studio model for simulating the impact of *n_R_* radial turns. In this case *n_R_* = 5. The tubing is modeled as Archimedean spiral whereby the beginning and the end are connected together giving a series connection of all *n_R_* turns. (**b**) shows a cross-section of the model in the *z-y*-plane of the exited differential transformer using a primary voltage of 1 V_PP_ at 155 kHz. The *x*-component of the resulting induced current density *J_S,x_* is color coded. The sample conductivity *κ* is 2 S/m. *d_S_* is the distance for the upper secondary coil *L_Sec1_* to the center of the sample and is 3.55 mm. *r*_0_ = 10 mm is the minimum inner radius of the spiral.

For simulating the behavior of the differential transformer and its dependence of the number of *n_R_* radial turns, we use the CST-EM Studio model described in Section 2.2. Therefore, a sample with a cross-sectional diameter of 4 mm was wrapped around the ferrite core in order to imitate the tubing used from the experimental section having an inside diameter *D_t,i_* of 4 mm and an outside diameter *D_t,o_* of 5.6 mm. The radial winding of the tubing can be described as an Archimedean spiral. To prevent the tubing from snapping off during later experimental investigations, the smallest inner radius of the spiral is limited to *r*_0_ = 10 mm. The model is illustrated in Figure 4a. With each additional radial turn *n_R_*, the radius increases by the outer diameter *D_t,o_* of the tubing and thus corresponds to the tightest possible winding radius of the tubing. In reality, the beginning and the end of the winding are connected together via the rest of the tubing system leading to a series connection of all *n_R_* turns. Since the length of the rest of the tubing usually depends on the application and is longer as shown in the first simulation, the impact of this will be investigated later. Figure 4b shows the *x*-component of the induced current density in a cross-section through the model in the *y-z*-plane, while the primary coil is excited with 1 V_PP_ at a frequency of 155 kHz. The induced current density of the *x*-component *J_S,x_* is color-coded. As can be seen, the current flows circularly around the ferrite core inside the tube. Due to the series connection of all turns, *J_S,x_* is approximately constant in all turns.

**Figure 5:**
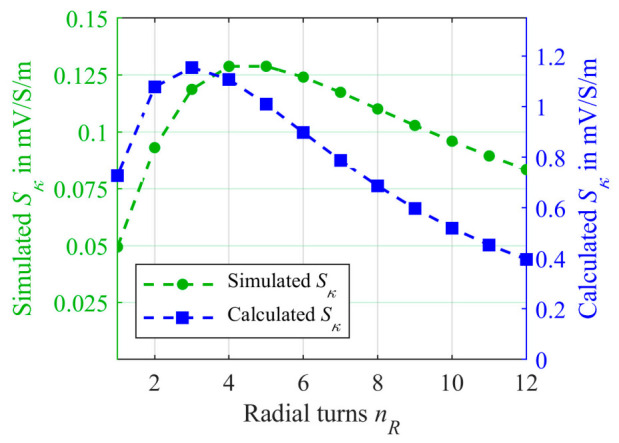
The simulated sensitivity *S_κ_* versus the number of radial turns *n_R_* using the CST-EM Studio model from Figure 4 is represented as green dots. *S_κ_* was determined using Equation (3) while the *κ* was changed from 1 S/m to 2 S/m. The blue squares represent the calculated sensitivity using the mathematical model from Equation (12).

The simulated sensitivity depending on *n_R_* was calculated according to Equation (3) and is represented as green dots in Figure 5.

As can be observed from the simulated sensitivity in Figure 5, the sensitivity initially improves as *n_R_* increases. However, after a maximum at 4 and 5 windings, the sensitivity declines again. This behavior of decreasing sensitivity after a certain maximum was already predicted in a mathematical model for a continually distributed sample with increasing outer sample radius in [45]. The reason for the decreasing sensitivity is an increasing penetration of both secondary coils with a uniform magnitude of the secondary magnetic flux density due to the rising mean radius of the sample. However, as also found in [45], such behavior could not be observed in the experiment, as the current distribution in a continuous sample is strongly non-linear in radial direction and decreases fast for higher radii, which is not included in this model. Hence, the effective mean sample radius does not rise proportional to the outer radius. As Figure 4 reveals, in the case of samples carried in a tubing, such a high decrease in current density in the radial direction cannot be observed. The series connection of all *n_R_* turns forces the current distribution to be nearly constant in all turns. In order to gain a more detailed understanding of sensitivity′ behavior as a function of *n_R_*, the mathematical model from [9] is adapted to samples carried in a tubing that is wrapped radially in the following section.

#### 3.1.2. Mathematical Model for Radial Winding

To obtain a mathematical model describing the behavior of the differential transformer, the primary magnetic flux penetrating the sample has to be calculated. The Biot–Savart law allows the calculation of the primary magnetic flux density *B_P_* along the *z*-axis at the position *z* = *d_PCB,C_* + *d_S_* [9]. *d_S_* is the distance between the center of the secondary coil *L_Sec1_* and the center of the sample in *z*-direction, as can be seen in Figure 4a. Moreover, the distance *d_PCB,C_* is used. It is the sum of the distance *d_PCB_* = 8 mm between the coils and the height *h_PCB_* = 1.5 mm of the PCB coils (*d_PCB,C_* = *d_PCB_* + *h_PCB_* = 9.5 mm). This means *d_PCB,C_* + *d_s_* is the distance between the center of the primary coil *L_P_* and the center of the sample. The distance *d_S_* = 0.5(*D_t,o_* + *h_PCB_*) = 3.55 mm. Biot–Savart′s law yields Equation (4) for the flux density *B_P_* caused by the coil *L_P_* excited with the current *I_P_* at the center of the sample:

(4) BPz=dPCB,C+dS =μ2rP,M2IPnPrP,M2+z232=μ2rP,M2IPnPrP,M2+dPCB,C+dS232.

Thereby it is simplified that all *n_P_* turns of the coil are located at the mean coil radius *r_P,M_*. *µ* describes the magnetic properties and consists of the product of *µ*_0_ (permeability of the free space) and *µ_r_* (the relative permeability of the material). As found in [9], *B_P_* can be considered almost uniformly distributed over the cross-sectional area in the *x-y*-plane of the ferrite core. In addition, as the permeability *µ* of the ferrite core (*µ_r,Fe_* = 300) is much higher by a factor of about 300 compared to the surrounding air, it can also be assumed in a simplified way that *B_P_* is zero outside the ferrite core, and thus is located solely inside the ferrite core. This enables the induced current *I_S_* inside the sample to be calculated using Faraday′s law. Assuming a sinusoidal primary field density *B_P_* with the angular frequency *ω* results in Equation (5):

(5) $$I_{S}\left(B_{P}\right)\;\approx -\frac{n_{R}}{Z_{S}}\frac{1}{dt}\iint_{A_{Fe}}^{}B_{P}\left(d_{PCB,C}+d_{M}\right)\;dA=-jn_{R}\frac{A_{t}\sigma_{S}}{l_{t}\left(n_{R}\right)}\pi r_{Fe}^{2}\omega B_{P}\left(d_{PCB,C}+d_{S}\right).$$

The total primary magnetic flux is, therefore, obtained by integrating *B_P_* over the cross-sectional area of the round ferrite core with the radius *r_Fe_*. Its time deviation d*t*^−1^ describes the induced voltage into one winding. For the sinusoidal *B_P_*, the time derivative can be substituted by *jω*. Since all *n_R_* turns are connected in series, this induced voltage has to be multiplied by the factor *n_R_*. However, the assumption made above of a disappearing flux density *B_P_* outside the ferrite core causes some error in particular for a large number of windings *n_R_*. Since the divergence of the field *B* is always zero, the field lines outside the ferrite core run in opposite directions of those inside the core. Thus, the outer windings in particular are penetrated by less magnetic flux than assumed by multiplication with the factor *n_R_* in Equation (5). Hence, it is to be expected that the induced voltage and hence the induced current *I_S_* according to Equation (5) overestimate the true current slightly. The induced current *I_S_* into the sample is obtained by multiplying the induced voltage with the reciprocal of the impedance *Z_S_*. *Z_S_* is proportional to *l_t_(n_R_)σ_S_^−1^A_t_^−1^*, where *l_t_(n_R_)* is the length of the tubing as a function of *n_R_*, *A*_t_ the cross-section of the tubing calculated as *A_t_ = π*(0.5*D_t,i_*)^2^ and *σ_S_* is the specific conductivity. *σ_S_* will be discussed in more detail later. For a first calculation of the induced current, it is assumed that *σ_S_* is approximately *κ*. To determine the length of the tubing, an Archimedean spiral is used to parameterize the radial winding as:

(6) $$S_{R}\left(\varphi \right)\;=\;\left(\begin{array}{c} x\\ y \end{array}\right)\;=\;\left(\begin{array}{c} \cos\left(\varphi \right)\\ \sin\left(\varphi \right) \end{array}\right)M\varphi ,$$

where *φ* is the angle of the tubing around the ferrite core (*z*-axis) and can be described using the integer number *n_R_* by *φ* = 2π*n_R_*. *M* describes the slope of the angle-dependent radius and is represented by *D_t,o_* (2π)^−1^. In addition to the spiral *S_R_*, a part for connecting the beginning and the end of the spiral together contributes to the total length *l_t_(n_R_)*. This can be divided into a winding-dependent part of the length *n_R_D_t,o_* in a radial direction and a constant part in the *z*-direction of 2∙10 mm. Consequently, Equation (7) enables the length to be calculated according [49]. Using the angle *φ*_0_ = 32π∙7^−1^ corresponds to the radius *r*_0_ + 0.5*D_t,o_* = 10 mm +2.8 mm, resulting in the mean radius of the innermost turn.

(7) $$l_{t}\left(n_{R}\right)\;=\int_{\varphi_{0}}^{\varphi_{0}+2\pi n_{R}}\;\sqrt{{\left(\frac{dS_{R,x}}{d\varphi }\right)}^{2}+{\left(\frac{dS_{R,y}}{d\varphi }\right)}^{2}}\;d\varphi +n_{R}D_{t,o}+2\cdot 10\;\mathrm{mm}\;=\frac{M}{2}{\left[\varphi \sqrt{1+\varphi^{2}}+\ln\left(\varphi +\sqrt{1+\varphi^{2}}\right)\right]}_{\varphi_{0}}^{\varphi_{0}+2\pi n_{R}}+n_{R}D_{t,o}+20\;\mathrm{mm}\;$$

**Figure 6:**
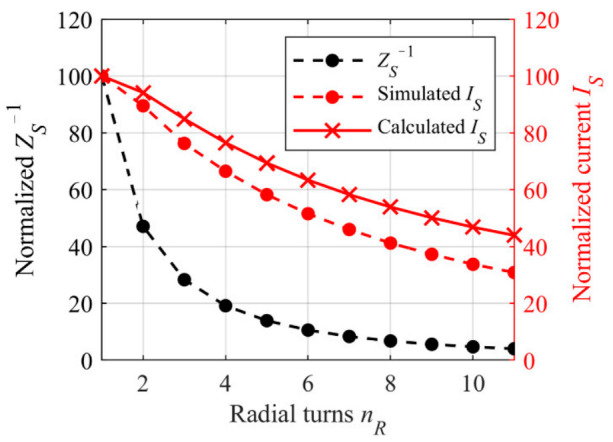
Comparision between the induced current *I*_S_ into the tubing system as a function of the radial turns *n_R_*, calculated according to Equation (5) (red crosses) and simulated using the CST-EM Studio model form Figure 4 (red dots) while *κ* = 2 S/m. For better comparability, both values are normalized to *I_S_*(*n_R_* = 1) (simulated: *I_S_(n_R_* = 1) = 3.66 µA; calculated: *I_S_*(*n_R_* = 1) = 4.8 µA) The reciprocal of the impedance *Z_S_* of the sample depending on *n*_R_ is shown as black dots also normalized to the initial value at *n_R_* = 1.

Now *Is*(*n_R_*) can be calculated according to Equation (5) for a sample conductivity of 2 S/m and is represented as red crosses (solid line) in Figure 6. For better comparability with the simulation, *I_S_* is normalized to *I_S_(n_R_* = 1) = 4.8 µA.

As can be seen, the induced current decreases with rising *n_R_*, although the induced voltage into the tubing increases by the factor *n_R_*. However, a closer look at Equation (5) reveals the proportionality of *I_S_* to *n_R_Z_S_*^−1^. As *Z*_S_ rapidly increases due to the increasing tubing length *l_t_*(*n_R_*), the reciprocal of *Z*_S_ drops fast. Figure 6 shows reciprocal of *Z_S_* depending on *n_R_*. *Z_S_*^−1^ is normalized to the initial value *Z_S_*^−1^(*n_R_* = 1) and is shown as black dots. The simulation with the CST-EM Studio Model confirms the decreasing induced current *I_S_*, as shown by the red dots (dashed line) in Figure 6. For better comparability with the calculated current, in this case the simulated current is also normalized to *I_S_*(*n_R_* = 1) = 3.66 µA (*κ* = 2 S/m). As expected, it can be observed that Equation (5) is slightly overestimating the induced current probably due to the simplification made for field *B_P_*. For low numbers of *n_R_* in particular, the calculated dependency of *I_S_* to the number of radial windings corresponds very well to the simulated dependency. For higher *n_R_*, an increasing deviation between the simulation and the calculation can be observed. This behavior was also anticipated, as described previously. The sample inside the tubing can now be considered as a coil with *n_R_* windings, driven by the induced current *I_S_* and producing the secondary magnetic field density *B_Sec_*. This field can now be calculated similar to *B_P_* by using Biot–Savart′s law. The radius can be regarded as the arithmetic mean of the inside radius *r*_0_ of the spiral and its outside radius *r*_0_ + *D_t,o_n_R_*. The Biot–Savart law yields Equation (8) for determining the magnetic flux density of the secondary magnetic field *B_Sec_* at the position *z = d_S_*, i.e., at the location of the upper secondary coil *L_Sec1_* as:

(8) BSec,LSec1z=dS =μ2r0+r0+Dt,onR22nRr0+r0+Dt,onR22+dS232 ISBP=−jμ2σSAt4 ltnRωπnPIPnR2r0+Dt,onR22 rFe2rP,M2r0+Dt,o nR22+dS232 rP,M2+dPCB,C+dS232.

Furthermore, the magnetic flux density of the secondary field *B_Sec_* at the location *2d_PCB,C_ + d_S_* is also of interest, since this corresponds to the position of the lower secondary coil *L_Sec2_*. This can be determined using:

(9) BSec,LSec2z=2dPCB,C+dS =μ2r0+r0+Dt,onR22nRr0+r0+Dt,onR22+2dPCB,C+dS232ISBP=−jμ2σSAt4 ltnRωπnPIPnR2r0+Dt,onR22rFe2rP,M2r0+Dt,onR22+2dPCB,C+dS232 rP,M2+dPCB,C+dS232.

The Faraday law yields Equation (10) for the output voltage *U_Sec_* at the differentially connected secondary coils *L_Sec1_* and *L_Sec2_*. Unlike Equation (5), when considering *U_Sec_* as terminal voltage, the sign has to be positive. Again, *B_Sec_* is simplified assumed to be evenly distributed inside the ferrite core and zero outside.

(10) USecnR =nSec1dt∬AFeBSec,LSec1−BSec,LSec2dA=jωπnSrFe2BSec,LSec1−BSec,LScec2=−j2μ24ω2nPnSecπ2rFe4rP,M2 rP,M2+dPCB,C+dS232IPσSAtltnRnR2r0+Dt,onR221r0+Dt,onR22+dS232−1r0+Dt,onR22+2dPCB,C+dS232=DÛPnR2r0+Dt,onR22ωε′−jκ+ωε″2ωLPltnRrP,M2+dPCB,C+dS232 (1r0+Dt,onR22+dS232−1r0+Dt,onR22+2dPCB,C+dS232)

In Equation (10), the conductivity σ_S_ can be separated by the proportionality ($\sigma_{S}\;\sim \;j\;\omega C_{S}+\kappa$), where *C_S_* describes the capacitive behavior of the sample [25]. C_S_ is proportional to the dielectric constant *ε*′ of the sample and the dielectric losses *ε*″ ($C_{S}\;\sim \;\varepsilon \prime -j\varepsilon \prime \prime$) [50]. As stated earlier the dielectric losses *ε*′′ can be neglected if the measuring frequency does not match a resonance in polarization [19,42]. In addition, the voltage *U_P_* exiting the coil *L_P_* and the coil impedance $j\omega L_{P}$, neglecting the small resistive part of the coil impedance, replaced the current *I_P_* of the primary coil ($I_{P}=\frac{{Û}_{P}}{j\sqrt{2}\omega L_{P}}$). To have a more compact term, *D* summarizes some values not considered further here and is defined as:

(11) $$D∶=\frac{\mu^{2}}{4}\pi^{2}n_{P}n_{Sec}r_{Fe}^{4}r_{P,M}^{2}\omega^{2}A_{t}.$$

For determination of the sensitivity of the differential transformer, Equation (3) is applied to Equation (10) leading to Equation (12).

(12) SκnR =DÛPnR2r0+Dt,onR222ωLPltnRrP,M2+dPCB,C+dM232(1r0+Dt,onR22+dS232−1r0+Dt,onR22+2dPCB,C+dS232)

For the length *l_t_(n_R_)*, Equation (7) has to be substituted into Equation (12). Plotting Equation (12) results in the blue curve in Figure 5. Thereby, the maximum sensitivity is 1.1 mV/S/m which is higher the simulated *S_κ,max_* of 128 µV/S/m. However, it should be noted that overestimation of the calculated *S_κ_* was expected as also the current was overestimated. Accordingly, the deviations may result from the simplifications made for Equation (12). More decisive is the course and the general dependence of *n_R_*, in order to identify the maximum sensitivity. As can be seen in Figure 5, both the calculated sensitivity according to Equation (12) and the simulated sensitivity have similar shape. After an initial increase in sensitivity by adding more turns *n_R_*, a maximum is reached followed by a decline for further *n_R_*. However, the calculation shows a slight shift of the maximum towards lower numbers of turns. Furthermore, the deviation between both curves increases with higher *n_R_*. As already mentioned, a possible reason for this could be the simplifications made, required for obtaining Equation (12). Nevertheless, Equation (12) is well suited to estimate the sensitivity. Moreover, it can be used to provide a good understanding of the behavior of the differential transformer by describing the dependence of *S_κ_* to *n_R_*. First, it shows increasing penetration of both secondary coils *L_Sec1_* and *L_Sec2_* of the same secondary magnetic flux density *B_Sec_* due to the increasing radius of the tubing spiral. Due to the differential setup, this has a negative effect in terms of sensitivity. This is revealed by the term in brackets in Equation (12). For higher *n_R_* this term becomes closer to zero. Secondly, the impact of the length *l_t_(n_R_)* of the tubing can be noted. At this point, we would like to mention that in order to determine the sensitivity *S_κ_* using the CST-EM Studio model, the conductivity *κ* of the sample must be varied in order to generate different output voltages *U_Sec_*. Therefore, *κ* was varied between 1 S/m and 2 S/m in the simulation model. Equation (3) can then be applied to the simulated voltages *U_Sec_* resulting in the simulated sensitivity. As can be seen from the mathematical model, the output voltage (Equation (10)) depends linearly on *κ*. Applying Equation (3) to Equation (10) results in a conductivity independent Equation (12). Therefore, it is not necessary to specify a conductivity range for the mathematical model at which the model has been investigated.

In the previous considerations, the length of the tubing *l_t_* was a function of the number of turns. Since the objective is using the existing tubing of the application, such as the extracorporeal circuit during dialysis treatment, the length is, therefore, independent of *n_R_* or at least shows less relative dependency. Hence, in the following section, the mathematical model is compared with measurements. In both cases, *l_t_* is left constant and independent of *n_R_*.

#### 3.1.3. Experimental Investigations for Radial Winding

The impact of the number of turns *n_R_* on the sensitivity and consequently on the precision of the differential transformer is investigated in this section experimentally. In the experimental tests, the tubing length is kept constant. As can be seen from Equation (5), the required length *l_t_* for the spiral grows rapidly with increasing *n_R_*. In addition to the spiral tubing part, tubing is needed to close the loop, and to pump the sample from a liquid container via a peristaltic pump to the differential transformer and back to the container again. The total tubing length is, therefore, 7.5 m. This fix length is also used for the mathematical model according to Equation (12). With this, about 15 turns can be realized. As in the previous section, the tubing is wrapped around the ferrite core in the form of an Archimedean spiral. All other tubing and spiral parameters, such as outer diameter *D_t,o_*, the inner diameter *D_t,i_* or the inside radius *r*_0_ of the spiral, are identical with those from the previous section.

**Figure 7:**
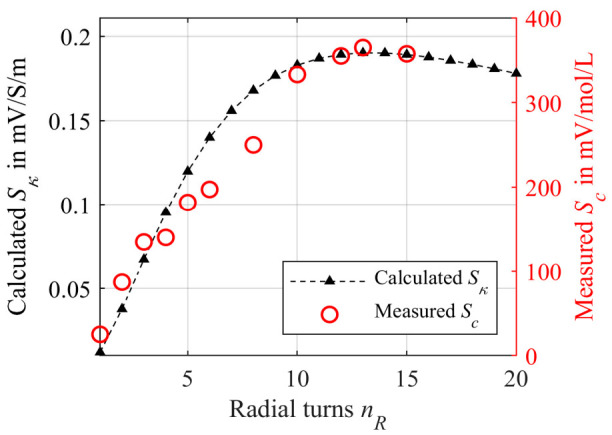
Calculated sensitivity S*κ* using the mathematical model according to Equation (12) with a constant tubing length *l_t_* = 7.5 m (black triangles). The red circles are the experimental measured sensitivities *S_c_* using the PCB differential transformer and NaCl solutions as sample. The differential transformer is driven with a primary voltage *U_P_* of 1 V_PP_ at 155 kHz. The tubing is wrapped around the ferrite core in radial direction with *n_R_* turns forming a planar multi-layer winding.

Considering the results obtained from the mathematical model with constant tubing length *l_t_* = 7.5 m, see Figure 7 (black triangles), and comparing this with Figure 5 (blue squares) with an *l_t_* depending on *n_R_* according to Equation (7), a significant impact of *l_t_* can be recognized. Increasing *l_t_* not only reduces the maximum sensitivity *S_κ,max_* from 1.1 mV/S/m to 0.19 mV/S/m. The maximum also shifts towards higher numbers *n_R_* = 13. After reaching the maximum, a gradual decline of the sensitivity can be noticed that can be attributed exclusively to the increasing penetration of both secondary coils with the same secondary field *B_Sec_*, as *l_t_* does not depend on *n_R_*. The reason for this is the growing radius of the spiral causing *B_Sec_* to propagate further in the *z*-direction [9,45], as described before.

**Figure 8:**
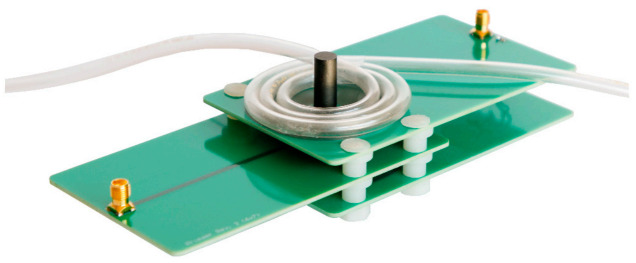
Photography of the PCB differential transformer for the experimental investigation with radial winding having *n_R_* turns of the tubing around the ferrite core forming a planar multi-layer winding. Here, it has three turns.

For the experimental validation, the PCB differential transformer from Section 2.1 is used. Figure 8 shows a photograph of this transformer with radially wrapped tubing.

The NaCl solutions of different concentrations *c* was pumped from the liquid container to the differential transformer and back to the container again. This allows the sensitivity *S_c_* to be calculated regarding to the concentration *c* as the input variable according to Equation (2). As can be seen in Figure 7 (red circles), the sensitivity increases at first until a maximum sensitivity *S_c,max_* of 364.9 mV/mol/L is detected at *n_R_* = 13. Subsequently, a decrease of *S_c_* is observed. The shape agrees well with the mathematical model, even if slight deviations can be noticed. As already mentioned, one possible reason could be the simplifications made for Equation (12). Nevertheless, the mathematical model can be used in a good approximation to estimate the behavior of the differential transformer. Comparing the sensitivity *S_c,max_* = 364.9 mV/mol/L achieved here with the sensitivity reached in earlier publications using a special flow chamber showing a maximum of 192 mV/mol/L, it is remarkable that the sensitivity is significantly higher when using the tubing. However, the flow chamber was designed for continuous in-line blood monitoring [9]. Thus, it was subject to the aforementioned design restrictions, i.e., to achieve hemocompatibility [45]. However, the question arises as to whether an *n_R_* of 13 is useful for the later application. For this, a length *l_t_* of the spiral of about 5 m (only the spiral part) would be required. This might conflict with shorter tubing required for dialysis. Thereby, it has to be investigated if a sufficient sensitivity and in particular, a sufficient precision is achieved even with fewer turns. As found in other publications [45], the noise of the output voltage *U_Sec_* is independent of the geometry of the sample. The same can be concluded here. In order to investigate the empirical standard deviation, the output signal of the differential transform was averaged 512 times by the oscilloscope Agilent DSO0104A. This results in a measuring value obtained approximately every 11 s. The empirical standard deviation was then determined from these measuring values. Averaging 512 times was chosen as a compromise between the improvement of the empirical standard deviation and time required for averaging. In the intended application of in-line blood parameter monitoring, a measuring value provided every 11 s could still be referred to as continuous monitoring. Of course, the previously executed averaging could also be reduced, meaning a measuring value is provided more frequently, but the empirical standard deviation is increased, or vice versa. For example, an empirical standard deviation of the output voltage *U_Sec_* of 31.42 µV is measured at *n_R_* = 2, while averaging in advance 512 times. At *n*_R_ = 6 it is 28.89 µV and at *n_R_* = 13 it is 30.74 µV. The corresponding sensitivities are *S_c_(n_R_* = 2) = 87.12 mV/mol/L, *S_c_(n_R_* = 6) = 197.3 mV/mol/L and *S_c_(n_R_* = 13) = 364.90 mV/mol/L. If the empirical standard deviation of the output voltage is divided by the respective sensitivity *S_c_*, the empirical standard deviation of the concentration is obtained. This is 0.36 mmol/L for *n_R_* = 2, 0.15 mmol/L for *n_R_* = 6 and just 0.08 mmol/L for *n_R_* = 13. As can be seen, higher sensitivity increases precision, since there is no systematic correlation between *n_R_* and the empirical standard deviation of *U_Sec_*. Comparing this with a commercially available blood gas analyzer, e.g., the GEM Premier 4000 from Werfen with a standard deviation of 0.6 mmol/L for sodium, the differential transformer outperforms this blood gas analyzer even for *n_R_* = 2 with 0.36 mmol/L [9,51] and has sufficient precision for medical applications. The required spiral length for *n_R_* = 2 is just about 25 cm. The reason for the lower empirical standard deviation of the differential transformer could be less susceptibility to electromagnetic interference than a potentiometric measurement as used in the blood gas analyzer.

The results show great potential for contactless measurement of the electromagnetic properties and in particular the conductivity of a sample running through tubing. Here, the tubing is wrapped around the ferrite core in a radial direction forming a planar multi-layer spiral with *n_R_* turns. The achieved sensitivity depends, among other things, on *n_R_*. In addition, the total length of the tubing and the mean radius of the spiral also affect the sensitivity. The shape of the sensitivity as a function of *n_R_* can be well estimated and predicted using the mathematical model found in Equation (12). In general, an increased sensitivity due to an optimized *n_R_* is associated with a higher precision of the differential transformer.

### 3.2. Longitudinal Winding

In this section, the behavior of the differential transformer for a longitudinal wound tubing with *n_L_* turns is investigated. At first, in Section 3.2.1 the behavior is analyzed using the CST-EM Studio model. Since finding a mathematical model for this case is much more complex compared to the radial winding and, therefore, no model exists, a more detailed discussion of the simulations takes place here. In Section 3.2.2 the simulation model is validated by experimental measurements using the PCB differential transformer. In addition, a closer look at the empirical standard deviation for this case is given.

#### 3.2.1. Numerical Simulations for Longitudinal Winding

**Figure 9:**
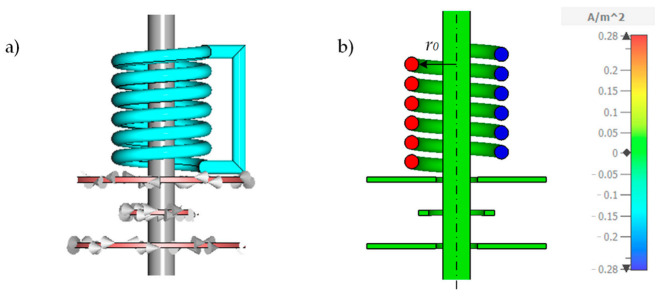
(**a**) Numerical CST-EM Studio model for simulating the impact of *n_L_* longitudinal turns forming a multi-layer helix. The inside radius *r*_0_ of the helix is 10 mm. Here, the helix has *n_L_* = 6 turns. The beginning and the end of the tubing are connected together giving a series connection of all *n_L_* windings. (**b**) Cross-section of the model in the *y-z*-plane of the exited differential transformer using a primary voltage of 1 V_PP_ at 155 kHz. The *x*-component of the resulting induced current density *J_S,x_* is color coded. The sample conductivity *κ* is 2 S/m.

As mentioned before, the tubing can also be wrapped around the ferrite core with *n_L_* turns longitudinal to the core. First, we simulate the impact of the number of *n_L_* turns on the sensitivity using the CST-EM Studio model from Section 2.2. Figure 9a shows the model with *n_L_* = 6. As before, the beginning and the end of the tubing are connected, meaning that all turns are in series. Figure 9b shows a cross-section of the simulation model with *n_L_* = 6 in the *y-z*-plane and the resulting induced current distribution *J_S,x_* inside the tubing color coded while the primary coil *L_P_* is excited with 1 V_PP_ at 155 kHz. 

Finding a mathematical model for describing the impact of the *n_L_* turns in the *z*-direction (longitudinal to the core) on the sensitivity *S_κ_* is much more complex compared to the radial winding. First, a different primary field density *B_P_* penetrates each turn, as *B_P_* decreases in the *z*-direction. Second, the distance between each turn of the winding and both secondary coils *L_Sec1_* and *L_Sec2_* depends on the turn itself, making it difficult to find an easy model describing the sensitivity. Therefore, in this section we will continue to improve the simulation model to match the experimental results.

Even if no mathematical model for the determination of the sensitivity is developed, the winding is parameterized in the following to calculate the length and thus the influence on the impedance. In the case of longitudinal winding, spiral *S_L_* can be represented as a helix using the parameter *φ* according to Equation (13). The inside radius of the helix is *r*_0_ = 10 mm and the mean radius of each turn is *r*_0_ + 0.5*D_t,o_*. The slope in the *z*-direction is defined by the outer hose diameter *D_t,o_* = 5.6 mm.

(13) $$s_{L}\left(\varphi \right)\;=\left(\begin{array}{c} x\\ y\\ z \end{array}\right)=\left(\begin{array}{c} \left(r_{0}+\frac{D_{t,o}}{2}\right)\cos\left(\varphi \right)\\ \left(r_{0}+\frac{D_{t,o}}{2}\right)\sin\left(\varphi \right)\\ D_{t,o}\frac{\varphi }{2\pi } \end{array}\right)$$

The length of the helix is given by Equation (14) [49].

(14) $$l_{L}\left(n_{L}\right)\;=2\pi \left(r_{0}+\frac{D_{t,o}}{2}\right)\sqrt{1+{\left(\frac{D_{t,o}}{\pi \left(r_{0}+\frac{D_{t,o}}{2}\right)}\right)}^{2}}n_{L}+\xi_{x}$$

As in the previous section, the beginning and the end of the helix are connected, increasing the total length. *ξ_x_* describes this additional length in Equation (14). In the first simulation model, we keep the additional length *ξ*_1_ as short as possible resulting in *ξ*_1_ = 20 mm + *n_L_D_t,o_*. However, as mentioned before, the total length of the tubing is usually longer and sometimes even independent of *n_L_*. In order to represent this in the simulation, we use a second simulation in which we insert a longer constant part of 800 mm, so that the change due to additional turns has less relative influence on the total length. In addition, the connection between the beginning and the end is made in such a way that the part *n_L_D_t,o_* is subtracted instead of being added, thus counteracting the winding-dependent increase according to Equation (14). This results in *ξ*_2_ = 800 mm − *n_L_D_t,o_*, which has to be substituted into Equation (14) for calculating the total length of the second simulation.

**Figure 10:**
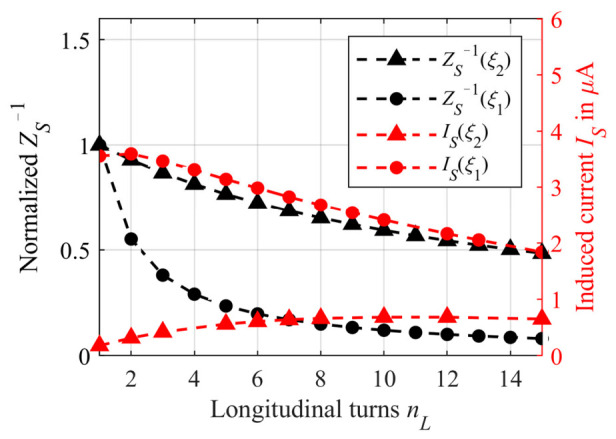
Reciprocal *Z_S_* ^−1^ for a tubing length calculated according to Equation (14) with *ξ*_1_ = 20 mm + *n_L_D_t,o_* (black dots) and *ξ*_2_ = 800 mm − *n*_L_*D_t,o_* (black triangles) normalized to *Z*_S_^−1^(*n_L_* = 1). In addition, the simulated induced current *I_S_* into the sample using the model from Figure 9 is shown as red dots for *ξ*_1_ = 20 mm + *n_L_D_t,o_* and as red triangles for *ξ*_2_ = 800 mm − *n_L_D_t,o_*. In both cases, the conductivity *κ* is 2 S/m.

Due to the series connection of all turns, the current distribution is approximately constant in all turns, as shown in Figure 10. The color gradient represents the *x*-component of the current density *J_S,x_*. By comparing the relative change in impedance reciprocal *Z_S_*^−1^ of the helix in Figure 10 for *ξ*_1_ (black dots) with that of the radial spiral in Figure 6 (black dots), it can be noticed that the impedance increase is less in the case of longitudinal winding. This is to be expected, since *Z_S_* is proportional to the length and the length increases less with each turn for the longitudinal case than in the radial case. The reciprocal of the impedance *Z_S_* in Figure 9 was normalized to *Z_S_*^−1^(*n_L_* = 1) in order to obtain the relative change. By taking a closer look at Equation (14), it can be noted that *Z_S_*^−1^ decreases approximately proportional to *n_L_*^−1^. However, due to the decreasing primary field density *B_P_* in increasing *z*-direction, it cannot be assumed for longitudinal winding that the induced voltage into the tubing increases proportionally to *n_L_*. Therefore, the induced current into the tubing also decreases, as can be seen from the simulated current *I_S_* in Figure 10 (red dots, *κ* = 2 S/m). Again, compared to the radial winding, the current *I_S_* decreases less with increasing *n_L_*. Since *I_S_* in Figure 10 is not compared to a calculated relationship as in Figure 6, *I_S_* is not normalized and the absolute simulated values are shown.

As expected, considering the relative change of *Z_S_*^−1^ for *ξ*_2_ (Figure 10, black triangles, also normalized to *Z_S_*^−1^ at the first turn), it can be seen, that the relative change is less compared to *ξ*_1_. As a result, the induced current *I_S_* into the sample initially increases with increasing *n_L_* (Figure 10, red triangles). However, due to the overall longer tubing compared to the shorter connection *ξ*_1_, the current is lower in absolute values.

**Figure 11:**
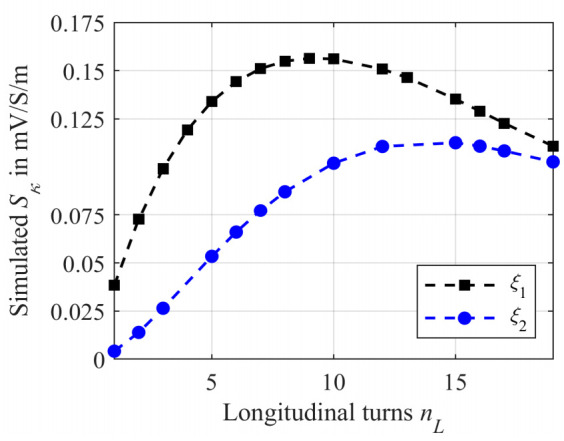
Simulated sensitivity *S_κ_* using the numerical CST-EM Studio model from Figure 9. The tubing is wrapped longitudinally to the ferrite core with *n_L_* turns and modeled as a helix having a radius *r*_0_ of 10 mm. The black squares represent the simulation using the connection *ξ*_1_ = 20 mm + *n_L_D_t,o_* between the beginning and the end of the helix and thus kept as short as possible. The blue dots represent the simulation for *ξ*_2_ = 800 mm − *n_L_D_t,o_* in Equation (14). *S_κ_* was determined using Equation (3) while the *κ* was changed from 1 S/m to 2 S/m.

Figure 11 shows the simulated sensitivity depending on *n_L_* for a tubing of length according to Equation (14) with *ξ*_1_ = 20 mm + *n_L_D_t,o_* (black squares) and *ξ*_2_ = 800 mm − *n_L_D_t,o_* (blue dots), connecting the beginning and the end of the tubing.

As can be noticed for both cases, initially the sensitivity increases with increasing number of windings and reaches a maximum. The maximum sensitivity *S_κ,max_* for *ξ*_1_ is located at *n_L_* = 9. Subsequently, the sensitivity decreases again. Furthermore, the maximum sensitivity is about 0.156 mV/S/m. Compared to radial winding with the maximum at *n_R_* = 4, the maximum for the shortest longitudinal winding method is at higher values of *n_L_*. 

For *ξ*_2_ the maximum is shifted towards *n_L_* = 15. Furthermore, due to the lower induced current *I_S_ S_κ,max_* is reduced to 0.113 mV/S/m. 

#### 3.2.2. Experimental Investigations for Longitudinal Winding

**Figure 12:**
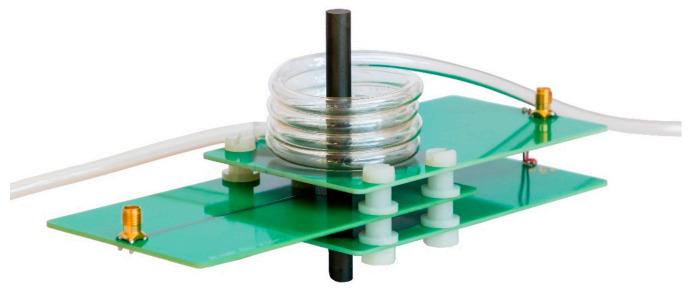
Photography of the PCB differential transformer for the experimental investigation with longitudinal winding having *n_L_* = 4 turns. The tubing around the ferrite core forms a helix.

In order to validate the findings from the simulations, we studied the dependency of the sensitivity *S_c_* by experimental investigations. As before, we used the same differential transformer and the sample solutions of different concentrations as for Section 3.1, allowing the sensitivity *S_c_* to be determined according to Equation (2). Figure 12 shows a photograph of the differential transformer with a longitudinal winding having four turns.

**Figure 13:**
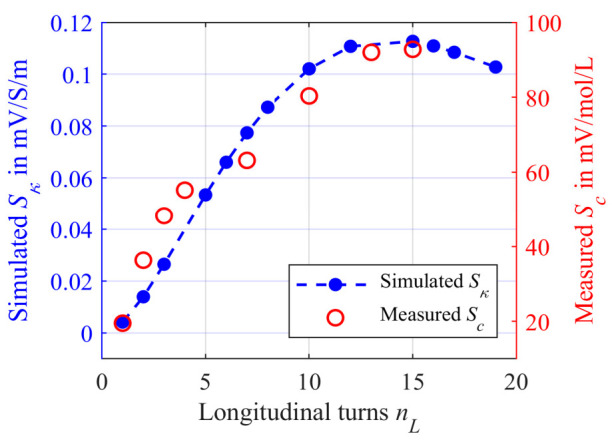
Simulated sensitivity *S_κ_* using the numerical CST-EM Studio model from Figure 9 (blue dots). The tubing is wrapped longitudinally to the ferrite core with *n_L_* turns and modeled as a helix having a radius *r*_0_ of 10 mm. The beginning and the end of the helix are connected together with a tubing an additional tubing of the length *ξ*_2_ = 800 mm − *n_L_D_t,o_*. *S_κ_* was determined using Equation (3) while the *κ* was changed from 1 S/m to 2 S/m. The red circles represent the measured sensitivity *S_c_* using the PCB differential transformer from Figure 12. The tubing was flushed with different concentrations *c* of a NaCl solution (*c* = 100 mmol/L to 150 mmol/L), so that the sensitivity could be determined according to Equation (2). The tubing length *l_t_* was constant in the experimental investigations at *l_t_* = 3.5 m.

Similar to the simulation model, we wrapped the tubing around the ferrite core as helix with a radius *r*_0_ of 10 mm. The total tubing length was kept constant at 3.5 m. This allows about 15 windings using the helix. The results of the measurements are represented by the red circles in Figure 13 and are compared to the second simulation with the additional tubing part *ξ*_2_ (blue dots). 

The measured sensitivity *S_c_* correlates very well with the simulated sensitivity *S_κ_*. A decrease in sensitivity cannot be observed until *n_L_* = 15. The maximum measured sensitivity is about 92.8 mV/mol/L. Compared to the radial winding with *S_c,max_* = 364.9 mV/mol/L, the maximum sensitivity *S_c,max_* is much lower. Thus, the radial winding is more effective in terms of sensitivity. A possible reason for this could be the decreasing field distribution of the primary field density in the *z*-direction. In addition, the turns also move further away from the secondary coil with each additional *n_L_*. Both effects may be the reason for a lower *S_c,max_*. However, the required tubing length for the longitudinal winding is lower.

As with the radial winding, an increasing sensitivity was found to be associated with an improved precision of the differential transformer. For example, the empirical standard deviation of the output voltage *U_Sec_* was measured as 30.47 µV at *n_L_* = 2, 30.73 µV at *n_L_* = 7, and 29.33 µV at *n_L_* = 15, while averaging in advance 512 times in 11 s. Again, no inherent correlation between *n_L_* and the empirical standard deviation of the output voltage can be found. The corresponding sensitivities are *S_c_*(*n_L_* = 2) = 36.32 mV/mol/L, *S_c_*(*n_L_* = 7) = 63.15 mV/mol/L and *S_c_*(*n_L_* = 15) = 92.8 mV/mol/L. The empirical standard deviation of the concentration is obtained by dividing the empirical standard deviation of the voltage by the respective sensitivity. Consequently, 0.84 mmol/L is calculated at *n_L_* = 2, 0.47 mmol/L at *n_L_* = 7 and 0.32 mmol/L at *n_L_* = 15. Again, the differential transformer outperforms the commercially available blood gas analyzer for *n_L_* ≥ 7 [9,51]. The required tubing length of *n_L_* = 7 is about 50 cm.

## 4. Conclusions

In this work, we have demonstrated a truly contactless approach to determine the electrical and dielectric properties of a sample carried in an electrical non-conductive tubing. Therefore, we used a differential inductive method based on a differential transformer. It has been shown that wrapping the tubing around the ferrite core of the differential transformer results in an increased sensitivity and thus improving precision. There are two basic winding options. The tubing can be wrapped with *n_R_* windings in the radial direction to the ferrite core forming a planar multi-layer spiral, or the tubing can be wrapped longitudinally to the ferrite core, i.e., in the *z*-direction with *n_L_* turns giving a helix. 

Wrapping the tubing in the radial direction has been found to be better in terms of sensitivity and precision compared to longitudinal winding. The highest sensitivity was achieved at *n_R_* = 13 turns with *S_c_*(*n_R_* = 13) = 364.90 mV/mol/L. As the number of turns does not affect the empirical standard deviation of the output voltage *U_Sec_*, a higher sensitivity is associated with a higher precision. For example, calculating the empirical standard deviation in concentration, while averaging *U_Sec_* 512 times in 11 s is just 0.08 mmol/L at *n_R_* = 13 and thus clearly outperforms a commercially available blood gas analyzer, e.g., the GEM Premier 4000 form Werfen with a standard deviation of 0.6 mmol/L for sodium. Even with *n_R_* = 2, a sensitivity *S_c_*(*n_R_* = 2) = 87.12 mV/mol/L can be measured, resulting in an empirical standard deviation in concentration of 0.36 mmol/L and thus outperforms the blood gas analyzer.

In conclusion, it was shown that the electromagnetic properties of samples guided in a tube can be determined with high sensitivity and also high precision. As a result, sensor-induced contamination of the sample is prevented, providing a major advantage in e.g., medical, biotechnological and chemical applications.

## Data Availability

The data presented in this study are available on request from the corresponding author.
